# Diametrically opposed sex‐specific effects of autistic traits on risk‐taking in poker

**DOI:** 10.1002/pcn5.70372

**Published:** 2026-07-07

**Authors:** Yosuke Eriguchi, Satoshi Eguchi, Hitoshi Kuwabara, Naoto Aoki, Miho Kuroda, Eisuke Sakakibara, Kiyoto Kasai, Yukiko Kano

**Affiliations:** ^1^ Department of Child Neuropsychiatry, Graduate School of Medicine The University of Tokyo Tokyo Japan; ^2^ Department of Child and Adolescent Neuropsychiatry Setagaya‐Yoga Clinic Tokyo Japan; ^3^ Professional Degree Program in Clinical Psychology, Graduate School of Clinical Psychology Teikyo Heisei University Tokyo Japan; ^4^ Department of Psychiatry Saitama Medical University Saitama Japan; ^5^ Department of Neuropsychiatry Sakura Hospital Aomori Japan; ^6^ Department of Psychology Den‐En Chofu University Kanagawa Japan; ^7^ Department of Neuropsychiatry, Graduate School of Medicine The University of Tokyo Tokyo Japan; ^8^ Department of Child Neuropsychiatry National Medical and Educational Consulting Center for Children Tokyo Japan

**Keywords:** autism spectrum disorder, avoidant rigidity, Bayesian inference, sex‐based differences, Texas Hold'em poker

## Abstract

**Aim:**

Autism spectrum disorder (ASD) clinical manifestations diverge significantly by sex, often leading to diagnostic delays in females. While social factors like camouflaging are recognized, the underlying computational mechanisms remain elusive. We investigated whether strategic decision‐making under incomplete information reflects these sex‐specific phenotypes through a Bayesian framework.

**Methods:**

Twenty poker‐naive adults (9 males, 11 females) with varying ASD traits played 100 hands of Texas Hold'em against a poker program. We employed generalized linear mixed models (GLMMs) incorporating interactions between sex and the Autism Diagnostic Observation Schedule, Second Edition (ADOS‐2), while controlling for full‐scale intelligence quotient (IQ), anxiety, and depression as covariates.

**Results:**

We identified a significant Sex × ADOS‐2 interaction. Males with higher ASD traits exhibited “aggressive rigidity”, characterized by increased betting and high‐risk bluffing (heavy investment with weak hands), reflecting a prior over‐precision that ignores negative feedback. Conversely, high‐trait females demonstrated “avoidant rigidity”, marked by extreme risk‐aversion and under‐betting even with strong hands. While higher IQ facilitated adaptive strategic resets (defensive folding after major losses) in females, this compensatory effect was neutralized in high‐trait males. These strategic biases remained independent of general anxiety and depression throughout the session, revealing a covert computational internalizing phenotype.

**Conclusion:**

This pilot study points toward a computational dimorphism: high‐magnitude aggression in males mirrors visible externalizing symptoms, whereas low‐magnitude avoidance in females aligns with covert internalizing symptoms. Identifying extreme risk‐aversion as a characteristic feature of the female ASD phenotype provides a novel, computationally informed framework to improve the identification of overlooked individuals.

## Graphical abstract

Sex‐specific strategic rigidity in autism spectrum disorder (ASD)

- **Male phenotype (aggressive rigidity)**: Persistent high‐risk bluffing despite weak hands.
- **Female phenotype (avoidant rigidity)**: Extreme risk‐aversion and failure to capitalize on strong hands.
- **Clinical impact**: Strategic decision‐making tasks identify invisible female ASD phenotypes often masked by internalizing symptoms.

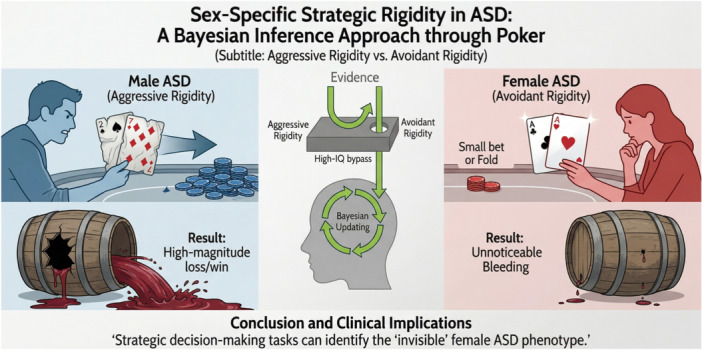

## INTRODUCTION

Autism spectrum disorder (ASD) encompasses a heterogeneous group of neurodevelopmental conditions characterized by atypical communication behaviors, repetitive interests, and altered sensory processing.^1^ Historically, ASD has been reported with a marked male dominance, often cited at a ratio of approximately 4:1.^2^, ^3^, ^4^, ^5^ However, recent large‐scale epidemiological data—such as a 2026 Swedish study of 2.7 million individuals—suggest that when diagnostic barriers are removed, the prevalence up to age 20 is nearly equivalent between sexes.^6^

Traditional diagnostic criteria and tools were developed based on male observations, often failing to capture the unique manifestations of ASD in females.^7^, ^8^, ^9^, ^10^ A key factor is social camouflaging or masking, where autistic women frequently mirror neurotypical interpersonal behaviors to meet cultural expectations, effectively obscuring their core difficulties.^11^, ^12^, ^13^ Furthermore, female interests (e.g., animals, literature^14^) may appear more conventional than those of males,^15^ leading clinicians to overlook their repetitive or intense nature.^16^ Consequently, autistic women are often diagnosed much later in life,^17^ or their symptoms are misattributed to secondary conditions such as anxiety, depression.^18^, ^19^ Crucially, understanding these unique clinical manifestations requires distinguishing them from general population baselines. In behavioral economics and gambling contexts, males are typically characterized as more risk‐prone than females.^20^, ^21^ This baseline asymmetry is further compounded by socialization and gender roles; traditional masculine roles often encourage aggressive risk‐taking under uncertainty, whereas feminine roles frequently reinforce avoidance of reputational loss.^22^, ^23^

However, rather than merely replicating these well‐established neurotypical sex differences, the critical challenge lies in elucidating how underlying autistic traits interact with, or diverge from, these baseline behavioral profiles. While clinical research has increasingly highlighted these phenotypic^24^, ^25^ and social disparities,^26^, ^27^ the underlying computational mechanisms that drive such ASD‐specific, sex‐divergent behavioral expressions remain poorly understood. Understanding these cognitive foundations is essential to move beyond surface‐level observations and develop sex‐sensitive diagnostic markers. A promising framework for this investigation is Bayesian inference, which models how the brain integrates internal prior expectations with external sensory evidence.^28^

Recent Bayesian theories have provided profound insights into ASD, typically characterizing it as an atypical balance in precision weighting—often an over‐reliance on immediate sensory details at the expense of flexible, high‐level priors.^29^, ^30^, ^31^ However, most existing Bayesian research has focused on low‐level sensory perception. There is a critical lack of research applying this framework to complex, high‐level strategic decision‐making, which requires the sophisticated integration of recursive beliefs and social uncertainty.^32^, ^33^

To bridge this gap, we employed Texas Hold'em—the globally dominant poker variant—as an experimental task. The strategic essence of poker demands advanced Bayesian inference under conditions of incomplete information^34^; players must constantly update their beliefs about hidden variables,^35^ such as an opponent's private cards and shifting behavioral types.^35^, ^36^ By analyzing how players with varying autistic traits navigate these strategic uncertainties and integrate feedback, this study seeks to explore whether the observed clinical dimorphism in ASD is reflected in distinct computational patterns of decision‐making.

## METHODS

### Ethics statement

We explained the purpose of this study to all participants, who subsequently provided written informed consent. The Ethics Committee of the University of Tokyo Hospital approved this study (Approval number: 12023).

### Study participants

Due to the intensive clinical evaluation required for each participant, this study was designed as an exploratory pilot investigation. Potential participants were recruited from the Developmental Disorders Inpatient Program, hosted at the Department of Neuropsychiatry at the University of Tokyo Hospital. The Developmental Disorders Inpatient Program provides one week of evaluation and psychoeducation for adults with possible ASD or attention‐deficit hyperactivity disorder. Potential participants underwent a comprehensive clinical evaluation, which included the Autism Diagnostic Observation Schedule, Second Edition (ADOS‐2),^37^ Wechsler Adult Intelligence Scale (WAIS‐IV)^38^ or Tanaka‐Binet Intelligence scale, State‐Trait Anxiety Inventory^39^ (STAI), and Center for Epidemiologic Studies Depression Scale^40^ (CES‐D). To ensure a uniform baseline experience, all recruited participants were Texas Hold'em novices.

### Experimental setting and recording

**Figure 1:**
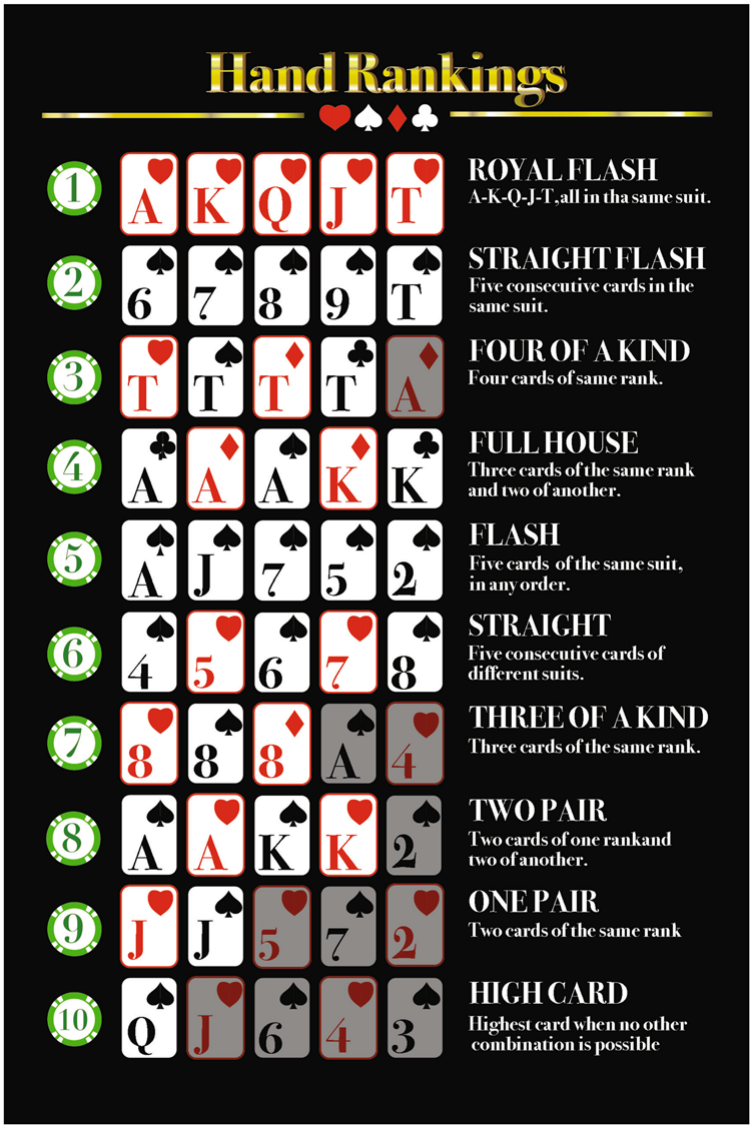
Hand rankings. The hands are shown from strongest (top) to weakest (bottom).

**Figure 2:**
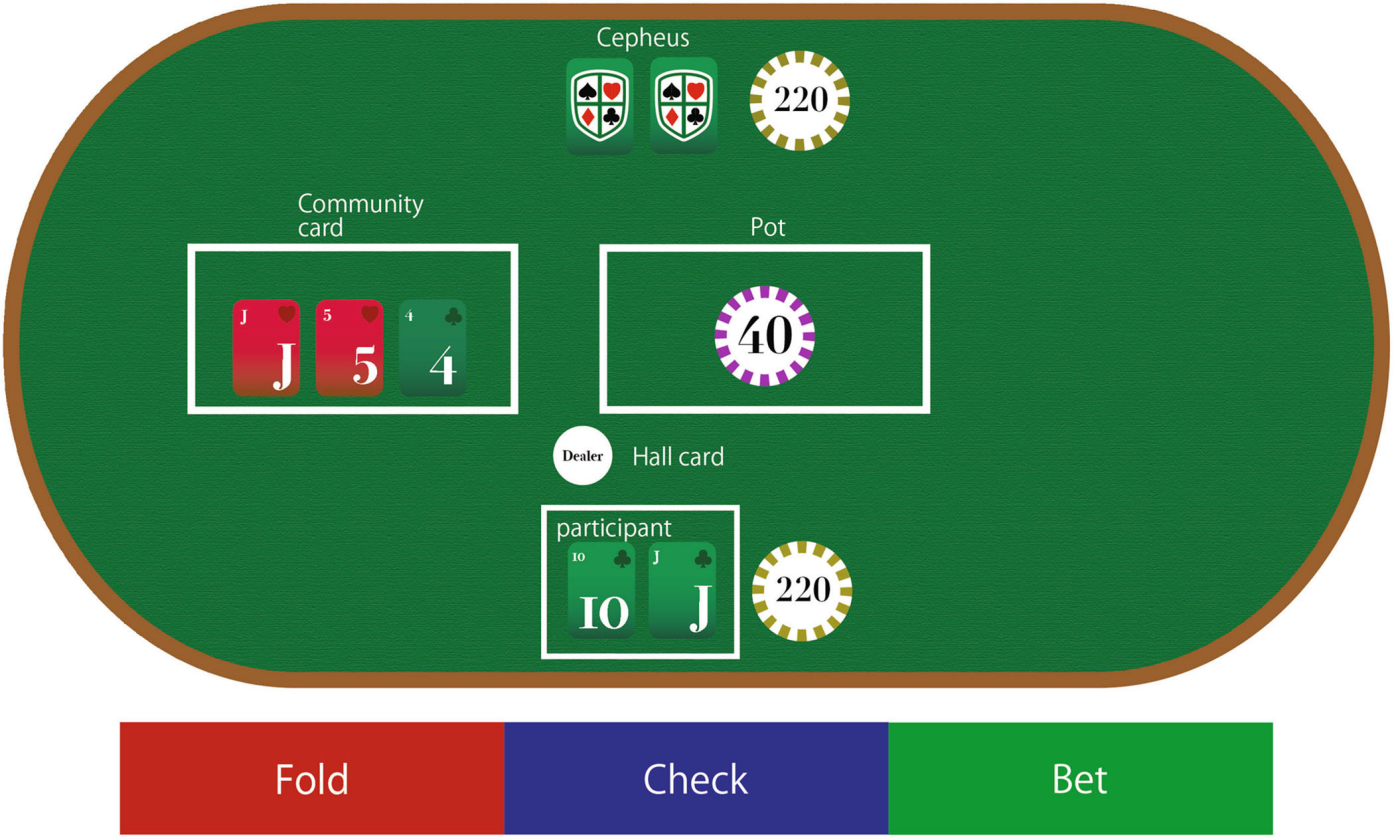
An example illustration of a board in Texas Hold'em. The lower middle white box shows the participants' cards. The upper left white box shows the community cards. The upper right white box shows the sum amount of money betted (pots). This illustration shows the community cards in the post‐flop round. Additional cards will show up in the turn and river rounds.

The participants received an explanation of the rules of Texas Hold'em using visual aids (Figure 1). They played against Cepheus,^41^ a computer agent employing a Game Theory Optimal (GTO) strategy, to ensure a static and non‐exploitative environment (Figure 2). The first 30 moves were regarded as training, and participant and Cepheus actions were recorded for the next 100 moves. The experiments were conducted in a quiet room. The participants received monetary compensation for their participation. The hand history data generated by the web‐based experiment platform systematically logged all participant actions and community cards. However, by system design, private hole cards were only recorded in the textual action log if a hand reached the showdown stage; cards forfeited during a fold were not captured in the raw data stream. Consequently, to ensure data uniformity, all downstream complex models were restricted to these showdown trials.

### Data processing

We counted the participants' actions, comprising betting, raising, calling, checking, and folding. According to previous study findings,^42^, ^43^ these actions were classified into three distinct categories: aggressive, consisting of betting and raising; passive, comprising calling and checking; and defensive, which entailed folding. ADOS‐2 sum scores were calculated by adding the scores from two domains: “Social Affect” and “Restricted and Repetitive Behaviors.” Hand outcomes were classified as winning or losing based on the net balance of exchanged chips.

### Statistical analyses

#### Action analysis

Action frequencies (bet, raise, call, check, and fold) were modeled using negative binomial regression to account for overdispersion and tied values. To identify sex‐specific behavioral phenotypes, we prioritized the Sex × ADOS‐2 interaction. To maximize statistical power given the sample size, a step‐down variable reduction procedure was applied. Age and full‐scale intelligence quotient (IQ) were retained as fundamental a priori covariates across all models. Psychiatric measures (STAI, CES‐D) were included as additional confounders only in the action and outcome analyses if they exhibited a marginal association (*p* < 0.10) in preliminary screenings. To prioritize model parsimony and avoid over‐parameterization, these psychiatric measures were excluded from the more complex subsequent mixed‐effects models. Results are reported as incidence rate ratios (IRRs).

#### Outcome analysis

To assess decision‐making intensity, we calculated mean showdown win/loss magnitudes, which were defined as the average number of chips exchanged during final confrontations. Non‐showdown outcomes were evaluated via mean fold‐induced win magnitude and mean post‐fold loss magnitude. These metrics utilized negative binomial models with an offset term for event frequency to estimate average stakes per decision. Finally, a linear model (LM) was applied to the final net balance to determine if strategic differences translated into overall financial disparities. In addition, to supplement the regression analysis, we defined substantial loss based on two thresholds of the final cumulative net balance (≤−500 and ≤−1000 chips) to identify individuals experiencing significant strategic collapse.

#### Hand strength analysis

Decision quality was quantified by calculating the percentile rank of each showdown hand via brute‐force simulation, comparing participants' hands against all 1081 possible combinations.

##### Mean percentile rank analysis

We employed a linear mixed‐effects model (LMM) to examine the influence of ADOS‐2 Total Score, Sex, and their interaction on mean hand strength. To account for the hierarchical structure of the data (multiple showdowns nested within participants), a random intercept for Participant ID was specified.

##### Strategic rigidity analysis

To quantify suboptimal decision‐making, we defined two complementary metrics of strategic failure: **high‐risk bluff**: a trial reaching a showdown with a bottom 30th percentile hand and a total investment exceeding the median pot size, representing extreme risk‐taking. **Under‐**betting (exploitation deficit): a trial reaching a showdown with a top 30th percentile hand and a total investment below the median pot size, representing a failure to maximize expected value. The probability of these behaviors was analyzed via generalized linear mixed‐effects models (GLMM) with a binomial distribution and logit link function.

#### Learning and adaptation effects

To distinguish stable behavioral traits from task acquisition or learning effects, we conducted two additional analyses. First, we estimated individual learning slopes by regressing net balance against trial number for each participant. Second, we performed sensitivity analyses focusing on the latter half of the session (hands 51–100) for both high‐risk bluffing and under‐betting frequencies to confirm the robustness of our primary findings.

#### Strategic adaptation following major losses

Adaptive capacity to negative feedback was investigated by analyzing behavioral shifts following a major loss (deficits in the top 25th percentile). We examined the probability of a Strategic Reset—defined as a low‐stake fold (pot size below median) in the subsequent trial. This captures the transition from high‐risk to defensive states. The probability of these resets was analyzed via a similar binomial GLMM.

Statistical significance was set at *p* < 0.05. All analyses were performed using R software (version 4.4.1).

## RESULTS

Between December 2018 and October 2021, 21 potential participants (9 males, 12 females) were enrolled. Owing to technical problems, we could not record one player's game; hence, 20 participants (9 males and 11 females) were included in the analysis. Participant characteristics are summarized in Table 1. Independent *t*‐tests (or Wilcoxon rank‐sum tests where appropriate) revealed no significant differences between the male and female groups in terms of age, IQ, or affective measures including STAI and CES‐D (*p* > 0.10). Although the ADOS‐2 total scores were numerically higher in males than in females, this difference was not statistically significant (*p* = 0.14), indicating equivalent baseline severity between sexes.

**Table 1:** Demographic and clinical characteristics of participants.

Variable	Total (*N* = 20)	Male (*n* = 9)	Female (*n* = 11)	Test of difference
	Mean (SD)	Mean (SD)	Mean (SD)	*p*‐Value
Age (years)	29.75 (10.26)	27.89 (9.68)	31.27 (10.93)	0.47
Full‐scale IQ	98.90 (15.84)	97.11 (19.97)	100.36 (12.35)	0.49
ADOS‐2 total score	9.40 (2.09)	10.22 (1.72)	8.73 (2.20)	0.14
STAI (anxiety)	58.00 (10.19)	56.62 (8.65)	59.00 (11.49)	0.61
CES‐D (depression)	26.11 (12.54)	24.25 (13.54)	27.45 (12.24)	0.60

*Note*: Values are presented as mean (standard deviation). Groups were compared using independent *t*‐tests (for normally distributed variables) or the Wilcoxon rank‐sum test (for non‐parametric data). No significant differences were observed between female and male groups across any demographic or clinical measures.

Abbreviations: ADOS‐2, Autism Diagnostic Observation Schedule, Second Edition; CES‐D, Center for Epidemiologic Studies Depression Scale; IQ, full‐scale intelligence quotient; STAI, State‐Trait Anxiety Inventory.

### Action analysis

For aggressive actions including betting and raising, as well as passive actions such as calling and checking, STAI and CES‐D scores did not meet these criteria and were thus excluded from the final regression models. Conversely, for fold actions, STAI scores significantly contributed to the model's explanatory power and were retained in the final analysis.

**Figure 3:**
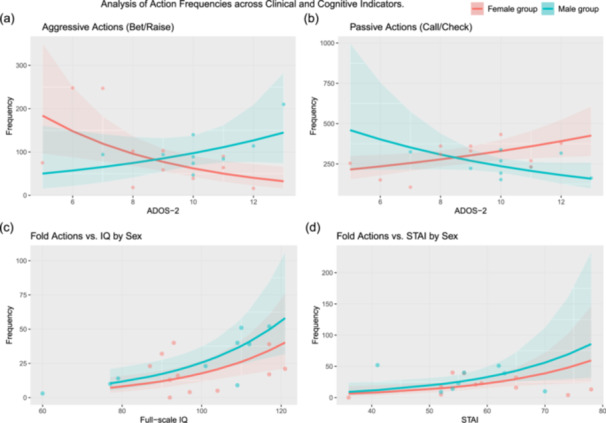
Analysis of action frequencies across clinical and cognitive indicators. (a) Aggressive actions (bet/raise): predicted frequencies of aggressive maneuvers show a significant Sex × Autism Diagnostic Observation Schedule, Second Edition (ADOS‐2) interaction (*p* = 0.007). In females, higher ADOS‐2 scores are associated with a marked reduction in aggression, whereas in males, higher traits drive an escalation in aggressive behavior. (b) Passive actions (call/check): a significant Sex × ADOS‐2 interaction (*p* = 0.013) demonstrates that the increased passivity observed in females with higher autistic traits is distinctly absent or reversed in their male counterparts. (c) Fold actions versus full‐scale intelligence quotient (IQ): in contrast to aggressive/passive actions, the frequency of folding is primarily predicted by general cognitive ability rather than autistic traits. Higher IQ significantly correlates with a higher frequency of folding (*p* = 0.002). (d) Fold actions versus State‐Trait Anxiety Inventory (STAI): anxiety levels show a significant positive correlation with folding frequency (*p* = 0.015), indicating that affective traits independently drive risk‐aversive behavior. Note: Solid lines represent predicted values from negative binomial regression models, and shaded areas indicate 95% confidence intervals. Raw data points are overlaid to show individual distributions.

The subsequent analysis revealed a highly significant Sex × ADOS‐2 interaction for aggressive actions (IRR = 1.42, *p* = 0.007; Figure 3a, Table 2). Specifically, higher autistic traits predicted reduced aggression in females (IRR = 0.81, *p* = 0.004) but a potent escalation in males. Similarly, a significant Sex × ADOS‐2 interaction was observed for passive actions (IRR = 0.80, *p* = 0.013; Figure 3b). Higher autistic traits predicted increased passivity in females (IRR = 1.09, *p* = 0.024), whereas this relationship was significantly inverted in males.

**Table 2:** Incidence rate ratios (IRRs) for aggressive, passive, and fold actions.

Predictor	Aggressive (bet and raise)	Passive (call and check)	Fold
	IRR [95% CI] (*p*)	IRR [95% CI] (*p*)	IRR [95% CI] (*p*)
(Intercept)	498.58 [62.32, 4184.46] (<0.001)	558.17 [115.82, 2811.12] (<0.001)	0.17 [0.00, 5.80] (0.323)
ADOS‐2	0.81 [0.68, 0.95] (0.004)	1.09 [1.01, 1.18] (0.024)	0.95 [0.81, 1.12] (0.559)
Sex (male)	0.05 [0.00, 0.50] (0.018)	6.34 [1.14, 37.10] (0.032)	0.24 [0.01, 8.62] (0.432)
IQ	1.00 [0.98, 1.01] (0.827)	1.00 [0.99, 1.01] (0.467)	1.04 [1.02, 1.07] (0.002)
Age	1.01 [0.98, 1.04] (0.432)	1.00 [0.98, 1.01] (0.568)	0.96 [0.93, 0.99] (0.023)
STAI	—	0.98 [0.96, 1.00] (0.085)	1.05 [1.01, 1.10] (0.015)
CES‐D	—	1.00 [0.99, 1.02] (0.591)	0.97 [0.94, 1.00] (0.067)
ADOS‐2 × Sex (male)	1.42 [1.12, 1.80] (0.007)	0.80 [0.67, 0.96] (0.013)	1.20 [0.83, 1.73] (0.335)

*Note*: IRRs were estimated using negative binomial regression models to account for the overdispersion of count data. Values in brackets [] indicate 95% confidence intervals, and values in parentheses () represent *p*‐values. An IRR > 1.00 indicates a positive association, while an IRR < 1.00 indicates a negative association with action frequency. STAI and CES‐D were included in the fold model.

Abbreviations: ADOS‐2, Autism Diagnostic Observation Schedule, Second Edition; CES‐D, Center for Epidemiologic Studies Depression Scale; IQ, full‐scale intelligence quotient; STAI, State‐Trait Anxiety Inventory.

In contrast to aggressive and passive actions, the frequency of folding was not driven by the Sex × ADOS‐2 interaction (*p* = 0.335). Higher IQ significantly predicted increased folding (IRR = 1.04, *p* = 0.002; Figure 3c), suggesting enhanced defensive inhibitory control. Furthermore, higher anxiety was associated with more frequent folding (IRR = 1.05, *p* = 0.015; Figure 3d), whereas depressive symptoms showed no significant effect. Age was also a significant predictor, with older participants folding less frequently (IRR = 0.96, *p* = 0.023).

### Outcome analysis

**Figure 4:**
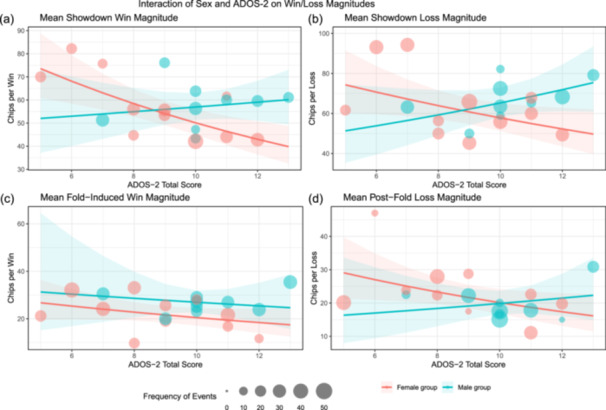
Interaction of sex and Autism Diagnostic Observation Schedule, Second Edition (ADOS‐2) on win/loss magnitudes. Scatter plots (bubbles) show the mean chips exchanged per single event for each participant, with bubble sizes representing the frequency of the respective events (i.e., total count of wins or losses). Regression lines and shaded areas indicate the predicted means and 95% confidence intervals derived from negative binomial models with an offset for event frequency. (a) Mean showdown win magnitude: average chips won in final confrontations. A significant Sex × ADOS‐2 interaction (*p* = 0.012) revealed that while females with higher autistic traits showed smaller win magnitudes, this trend was reversed in males, who maintained higher stakes in showdowns. (b) Mean showdown loss magnitude: average chips lost in final confrontations. Similar to win magnitudes, a significant Sex × ADOS‐2 interaction was observed (*p* = 0.018), reflecting divergent risk‐taking phenotypes between sexes. (c) Mean fold‐induced win magnitude: average chips won when the opponent folded. No significant interaction or main effects of ADOS‐2 were observed, suggesting that gains from opponent withdrawal were relatively stable across groups. (d) Mean post‐fold loss magnitude: average chips forfeited when the participant folded. In contrast to showdown outcomes, no significant Sex × ADOS‐2 interaction was observed for the magnitude of chips forfeited upon folding (*p* = 0.46). In sex‐stratified analyses, ADOS‐2 scores were not significantly associated with post‐fold loss magnitudes in either females (*p* = 0.103) or males (*p* = 0.486).

A significant Sex × ADOS‐2 interaction was observed for both mean showdown win magnitude (IRR = 1.10, *p* = 0.012; Table 3, Figure 4a) and mean showdown loss magnitude (IRR = 1.10, *p* = 0.018; Figure 4b). Among females, higher ADOS‐2 scores were associated with a significant decrease in the magnitude of chips won or lost per showdown, indicating a transition toward a low‐stakes, cautious strategy. In contrast, this trend was significantly reversed in males; the positive interaction term suggests that high‐trait males maintained or engaged in higher‐stakes showdowns, mirroring their increased frequency of aggressive actions.

**Table 3:** Incidence rate ratios (IRRs) for outcome magnitudes per event.

Outcome metric	Showdown win	Showdown loss	Fold‐induced win	Post‐fold loss
	IRR [95% CI] (*p*)	IRR [95% CI] (*p*)	IRR [95% CI] (*p*)	IRR [95% CI] (*p*)
ADOS (female)	0.93 [0.89, 0.97] (<0.001)	0.95 [0.91, 1.00] (0.036)	0.95 [0.88, 1.02] (0.134)	0.94 [0.88, 1.02] (0.103)
Sex (male)	0.44 [0.21, 0.92] (0.029)	0.42 [0.19, 0.94] (0.037)	1.04 [0.22, 5.08] (0.961)	0.54 [0.13, 2.32] (0.417)
IQ	1.00 [1.00, 1.00] (0.96)	1.00 [0.99, 1.00] (0.40)	1.00 [0.99, 1.01] (0.940)	1.00 [0.98, 1.01] (0.573)
Age	1.00 [1.00, 1.01] (0.35)	1.00 [1.00, 1.01] (0.23)	1.00 [0.98, 1.01] (0.627)	1.01 [0.99, 1.03] (0.307)
STAI	—	—	0.98 [0.97, 1.00] (0.086)	0.98 [0.96, 1.00] (0.084)
CES‐D	—	—	1.01 [1.00, 1.03] (0.094)	1.01 [1.00, 1.03] (0.077)
ADOS × Sex	1.10 [1.02, 1.18] (0.012)	1.10 [1.02, 1.20] (0.018)	1.02 [0.87, 1.20] (0.774)	1.06 [0.91, 1.22] (0.486)

*Note*: Magnitudes of chip gains and losses were modeled using negative binomial regression with an offset term for event frequency. Showdown win magnitude represents the average chips gained per victory, while showdown loss magnitude and post‐fold loss magnitude reflect the average cost of confrontations and defensive retreats, respectively.

Abbreviations: ADOS, Autism Diagnostic Observation Schedule; CES‐D, Center for Epidemiologic Studies Depression Scale; IQ, full‐scale intelligence quotient; STAI, State‐Trait Anxiety Inventory.

Mean fold‐induced win magnitude did not show significant associations with ADOS‐2 traits or sex (Figure 4c). Similarly, for mean post‐fold loss magnitude, which represents the chips forfeited upon folding, the Sex × ADOS‐2 interaction did not reach statistical significance (*p* = 0.486). In sex‐stratified observations, no significant association was found between ADOS‐2 scores and chips forfeited per fold for either females (*p* = 0.103) or males (*p* = 0.486; Figure 4d).

Although higher anxiety, as measured by the STAI, and depressive symptoms, captured by the CES‐D, were included based on our selection criteria (*p* < 0.10), neither factor exhibited a statistically significant impact on fold‐related outcomes (all *p* > 0.05). Finally, IQ and age showed no significant impact on these fold‐related magnitudes.

Regression analysis for the final net balance revealed no significant main effects for ADOS‐2 total score (*p* = 0.825; Supporting Information S3: Table 1) or Sex (*p* = 0.184), nor a significant Sex × ADOS‐2 interaction (*p* = 0.218).

To further investigate the strategic vulnerabilities that may be obscured by group mean differences, we performed an exploratory analysis of the distribution of final net balances. Although no statistically significant differences between sexes were observed in the frequency of severe cumulative losses, as evaluated by Fisher's exact test (*p* = 0.37 for ≤−500 chips; *p* = 0.22 for ≤−1000 chips), individual trajectories shown in Supporting Information S1: Figure 1 provided preliminary observations for future investigation. For instance, while three female participants (27.3%) reached losses exceeding −1000 chips, no male participants reached this threshold. These preliminary observations may suggest a potential vulnerability to continuous strategic collapse, or bleeding, in a subset of individuals, which warrants further replication in larger samples to determine if sex‐specific patterns exist.

### Hand strength analysis

#### Mean percentile rank analysis

Regarding the average hand strength at showdown, the LMM did not reveal a significant Sex × ADOS‐2 interaction (*p* = 0.305; Table 4, Supporting Information S2: Figure 2b).

**Table 4:** Linear mixed model (LMM) analysis of hand strength percentile at showdown.

Predictor	Estimate	Std. error	df	*t*‐Value	*p*‐Value
(Intercept)	53.29	9.5	16.3	5.61	<0.001
ADOS‐2 total (female)	−0.55	0.66	14.98	−0.83	0.419
Sex (male group)	15.95	11.31	14.18	1.41	0.18
IQ	0.03	0.07	14.62	0.38	0.707
Age	−0.11	0.11	11.38	−1.05	0.317
ADOS‐2 × Sex	−1.21	1.14	14.01	−1.07	0.305

*Note*: This table presents the effects of ADOS‐2 total scores, IQ, and age on the relative hand strength (percentile rank) during showdowns, stratified by sex. The percentile rank was calculated using a brute‐force simulation (*n* = 1081 combinations per community card set), where a lower percentile indicates a higher propensity for risk‐taking (i.e., reaching a showdown with an objectively weaker hand). IQ and age were included as covariates to control for general cognitive ability and age‐related effects. *N* = 1029 trials across 20 participants.

Abbreviations: ADOS‐2, Autism Diagnostic Observation Schedule, Second Edition; IQ, full‐scale intelligence quotient.

#### Strategic rigidity analysis

**Figure 5:**
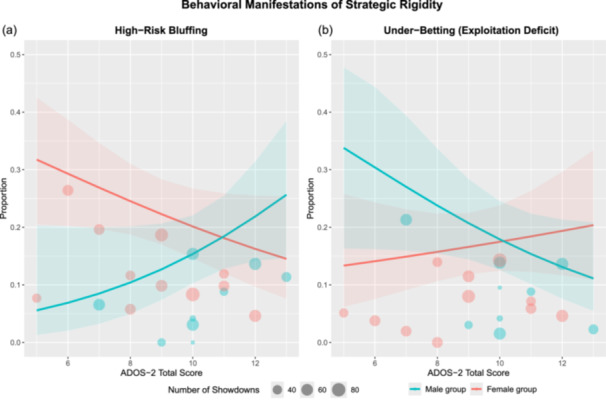
Behavioral manifestations of strategic rigidity. Each bubble represents an individual participant, with the size proportional to the number of showdowns. Solid lines indicate predicted probabilities from the mixed‐effects logistic regression model, and shaded areas represent 95% confidence intervals. (a) The occurrence of high‐risk bluffs. The *y*‐axis shows the proportion of hands identified as high‐risk bluffs (bottom 30% hand strength with above median pot). The distinct X‐shaped interaction illustrates that higher autism spectrum disorder (ASD) traits correlate with increased strategic recklessness in males, but increased caution in females. (b) The occurrence of under‐betting (exploitation deficit). The *y*‐axis shows the proportion of hands identified as under‐betting (top 30% hand strength with below median pot). While the Sex × Autism Diagnostic Observation Schedule, Second Edition (ADOS‐2) interaction for the entire session was not statistically significant (*p* = 0.061), sensitivity analysis focusing on the latter half of the session (hands 51–100) revealed a significant interaction (*p* = 0.012, see Supporting Information S5: Table 3).

The high‐risk bluff analysis revealed a significant Sex × ADOS‐2 interaction on the probability of engaging in reckless aggression (odds ratio [OR] = 1.44, *p* = 0.014; Table 5, Figure 5a). While the association between autistic traits and bluffing probability did not reach statistical significance in females across the full session (*p* = 0.074; however, see the 3.4 section for its temporal manifestation), this relationship was significantly reversed in males. Specifically, higher ADOS‐2 scores in male participants were associated with a significant increase in high‐risk bluffing (OR = 1.25, calculated from the interaction), suggesting a distinct aggressive rigidity in males compared to the non‐significant or more passive patterns observed in females. This strategic bias was independent of IQ (*p* = 0.366), suggesting that the aggressive prior in ASD males is a distinct phenotypic feature rather than a result of general cognitive factors.

**Table 5:** Logistic regression analysis of high‐risk bluffing probability.

Predictor	Odds ratio (OR)	95% CI	*z*‐Value	*p*‐Value
(Intercept)	0.49	[0.05, 4.39]	−0.637	0.524
ADOS‐2 total (female)	0.87	[0.75, 1.01]	−1.787	0.074
Sex (male group)	0.02	[0.00, 0.35]	−2.646	0.008
IQ	0.99	[0.97, 1.01]	−0.903	0.366
Age	1.02	[1.00, 1.05]	1.714	0.087
ADOS‐2 × Sex	1.44	[1.08, 1.93]	2.468	0.014

*Note*: A “high‐risk bluff” was defined as a showdown reached with a hand strength in the bottom 30th percentile combined with a total investment exceeding the median pot size. This analysis evaluated the probability of such reckless commitment using a generalized linear mixed model (GLMM) with a binomial distribution and a logit link function.

Abbreviations: ADOS‐2, Autism Diagnostic Observation Schedule, Second Edition; IQ, full‐scale intelligence quotient.

Regarding under‐betting, which reflects an exploitation deficit, the analysis of the entire 100‐hand session revealed that the Sex × ADOS‐2 interaction did not reach the significance threshold (*p* = 0.061; Table 6, Figure 5b). Instead, IQ emerged as a robust and significant predictor (OR = 0.97, *p* = 0.005); higher IQ was associated with a lower probability of under‐betting. These results suggest that, at least when considering the session as a whole, the specific ability to translate hand strength into profitable action is significantly influenced by general cognitive resources. Explicitly incorporating this baseline cognitive capacity highlights its critical role in modulating tactical adjustments under uncertainty, providing essential context for distinguishing general cognitive effects from ASD‐specific traits. This further suggests that higher cognitive capacity may serve as a critical factor in overcoming passive strategic failures, the temporal dynamics of which are subsequently explored through the sensitivity analysis in the 3.4 section.

**Table 6:** Mixed‐effects logistic regression analysis of under‐betting probability (exploitation deficit).

Predictor	Odds ratio (OR)	95% CI	*z*‐Value	*p*‐Value
(Intercept)	0.41	[0.03, 5.40]	−0.68	0.497
ADOS‐2 total (female)	1.07	[0.89, 1.28]	0.729	0.466
Sex (male group)	13.6	[0.97, 189.86]	1.941	0.052
IQ	0.97	[0.95, 0.99]	−2.834	0.005
Age	1.01	[0.99, 1.04]	0.924	0.356
ADOS‐2 × Sex	0.77	[0.59, 1.01]	−1.877	0.061

*Note*: “under‐betting” (or “exploitation deficit”) was defined as a hand in which a participant held superior strength (top 30th percentile) but failed to extract value, resulting in a total pot size below the median. This analysis evaluated the probability of such missed opportunities using a generalized linear mixed model (GLMM) with a binomial distribution and a logit link function. *N* = 1029 observations from 20 participants.

Abbreviations: ADOS‐2, Autism Diagnostic Observation Schedule, Second Edition; IQ, full‐scale intelligence quotient.

### Assessment of learning and adaptation effect

Individual‐level analysis revealed that only one participant, representing 5% of the sample, showed a statistically significant improvement in financial outcomes over the 100‐hand session (*B* = 0.317, *p* = 0.0242), indicating an absence of systematic financial learning across the cohort. However, sensitivity analyses focused on the latter half of the session, specifically hands 51–100, further clarified the underlying strategic phenotypes. In this period, where initial task‐familiarization effects were minimized, significant Sex × ADOS‐2 interactions emerged for both high‐risk bluffing (OR = 1.68, *p* = 0.016; Supporting Information S4: Table 2) and under‐betting (OR = 0.65, *p* = 0.012; Supporting Information S5: Table 3). These results suggest that while participants did not learn to improve their profit, the reduction of early‐session noise allowed their stable cognitive phenotypes to manifest more clearly. This supports the interpretation that the observed patterns suggest ingrained diagnostic traits rather than transient acquisition effects.

### Strategic adaptation following major losses

**Figure 6:**
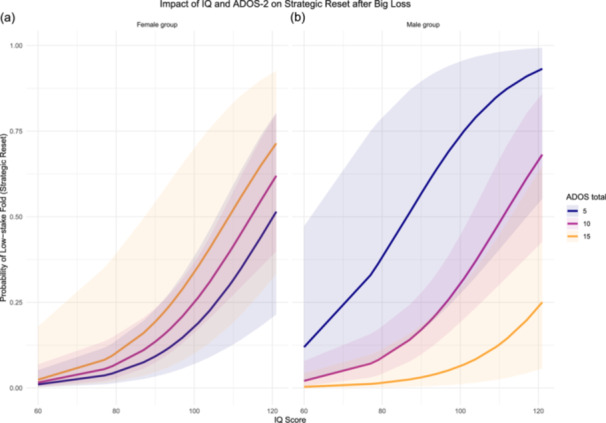
Impact of full‐scale intelligence quotient (IQ) and Autism Diagnostic Observation Schedule, Second Edition (ADOS‐2) on strategic reset after big loss. In females, IQ consistently promotes the probability of adaptive low‐stake folding following major losses. In contrast, for males, higher ADOS‐2 scores neutralize this intelligent error‐correction, leading to a state of strategic rigidity regardless of cognitive ability.

The GLMM revealed a complex interplay between cognitive ability and autistic traits in predicting strategic adaptation. A significant main effect of IQ was observed (OR = 1.079, *p* < 0.001; Table 7), indicating that higher general intelligence generally promotes the shift to defensive strategies following a major loss. Age showed a significant negative correlation with strategic resets (OR = 0.926, *p* = 0.002), suggesting that older participants were less likely to revert to immediate low‐stake folding. We found a significant Sex × ADOS‐2 interaction (OR = 0.633, *p* = 0.031). In female participants, strategic resets were primarily driven by IQ, regardless of ADOS‐2 scores. However, in male participants, the positive influence of IQ on strategic adaptation was significantly attenuated by higher ADOS‐2 scores (Figure 6). For high‐trait male participants, the compensatory effect of intelligence was effectively neutralized, leading to a state of strategic rigidity despite high cognitive capacity.

**Table 7:** Results of generalized linear mixed model (GLMM) predicting strategic reset following major losses.

Predictor	Odds ratio (OR)	95% CI	*z*‐Value	*p*‐Value
(Intercept)	0.001	[0.00, 0.02]	−4.027	<0.001
ADOS‐2 total	1.089	[0.86, 1.38]	0.706	0.48
Sex (male)	127.1	[1.96, 8256.2]	2.275	0.023
IQ	1.079	[1.04, 1.12]	4.239	<0.001
Age	0.926	[0.88, 0.97]	−3.101	0.002
ADOS‐2 × Sex (male)	0.633	[0.42, 0.96]	−2.154	0.031

*Note*: A strategic reset was defined as executing a low‐stake fold (in a pot below median size) in the trial immediately following a major loss (deficits in the top 25th percentile), representing an adaptive shift from high‐risk to defensive play. The model included participant ID as a random intercept. Statistical significance was estimated using Satterthwaite's method. *N* = 293 observations from 20 participants.

Abbreviations: ADOS‐2, Autism Diagnostic Observation Schedule, Second Edition; IQ, full‐scale intelligence quotient.

## DISCUSSION

Our study provides evidence that ASD traits characterize the Bayesian inferential process in poker, manifesting as sex‐specific patterns of strategic rigidity. This is supported by an integrated analysis of action frequencies, outcome magnitudes, and bluffing propensity, which reveals that the ASD strategic phenotype is characterized by a stable, directional bias that remains remarkably insensitive to environmental feedback.

In male participants, higher autistic traits were associated with a pronounced “aggressive rigidity”. This behavioral escalation, elucidated by our high‐risk bluff analysis, revealed that high‐ADOS males persistently committed to large investments despite holding objectively weak hands. Critically, these aggressive actions did not translate into increased profit, suggesting that the primary driver was an internal strategic prior rather than the objective probability of winning. This aligns with a state of prior over‐precision, where an internal offensive drive blunts sensitivity to likelihood signals, such as hand strength and opponent counter‐signals, remaining remarkably stable over 100 trials without task‐based learning.

In contrast, females with higher autistic traits exhibited an “avoidant rigidity”, characterized by extreme risk‐aversion and significantly fewer bets. While this strategy was intended to limit risk‐exposure, it led to smaller win magnitudes and a failure to capitalize on strong hands, resulting in suboptimal outcomes. Our exploratory analysis of final net balances further illuminated the cost of this avoidance. While a subset of females maintained a relatively stable balance, others exhibited a disproportionately high frequency of severe cumulative losses compared to the male group. Importantly, the observed strategic patterns remain robustly associated with autistic traits even when accounting for psychological distress factors, including anxiety and depression. While less overt than male externalizing aggression,^44^, ^45^ it is consistent with a diminished capacity for adaptive updating of internal strategic models based on winning probabilities. Specifically, once an avoidant strategy begins to fail against a consistent opponent, the inability to reset or take calculated risks leads to a state of strategic bleeding,^46^ where small, incremental losses accumulate into a significant terminal deficit.

Our findings reveal a critical phenotypic dissociation between general affective anxiety and ASD‐specific strategic avoidance. While higher STAI scores predicted frequent folding, general anxiety had no impact on aggressive or passive actions, nor did it modulate showdown magnitudes. This contrast suggests that the avoidant rigidity observed in high‐trait females is computationally distinct from the behavior driven by baseline anxiety. Clinically, folding represents a defensive yet active decision to truncate risk. In contrast, the female ASD phenotype was uniquely characterized by increased passive actions coupled with a prominent reduction in showdown magnitudes. Rather than executing a strategic exit via folding, the baseline phenotype associated with higher female ASD traits during routine gameplay manifested as a reliance on passive continuation; actions that neither assert strategic control nor protect the balance from erosion. This pattern suggests a state of decision‐deferred avoidance under uncertainty, rather than an active risk‐mitigation strategy. Consequently, by dissociating active risk truncation from passive non‐decision, the game‐theoretic framework of poker demonstrates that passive strategies may reflect a distinct inclination toward decision avoidance rather than generalized anxiety alone, thereby capturing these complex behavioral expressions at a higher resolution than traditional self‐report instruments. Crucially, because this passive avoidance showed no association with affective distress, it underscores a distinct, covert internalizing phenotype unique to the cognitive architecture of female ASD, rather than a mere byproduct of general anxiety. Because this latent phenotype easily mimics or co‐occurs with general anxiety symptoms, it is frequently misattributed to primary anxiety disorders,^47^, ^48^, ^49^ directly contributing to the severe diagnostic delays typically experienced by females with ASD.

Given the constraints described in the Methods, whether general anxiety directly interacts with these higher level strategic rigidities remains an open question. Intriguingly, while female ASD traits—characterized by calling and checking—appear strictly dissociated from anxiety‐driven folding during routine gameplay, these profiles may partially overlap in high‐stress scenarios. Specifically, our finding that strategic resets following a major loss—which demands immediate, defensive folding—were modulated by a sex and ASD trait interaction suggests that acute, loss‐induced distress might converge with baseline anxiety mechanisms in a context‐dependent manner.

Our results highlight that the interaction of sex and ASD traits—rather than biological sex alone—governs strategic bias under uncertainty. While socialization and compensatory camouflaging^11^ may shape the initial safety‐first prior in females, the remarkable stability of these patterns against GTO feedback underscores that the failure to update this prior template is robustly driven by ASD‐related rigidity rather than baseline sex differences.

**Figure 7:**
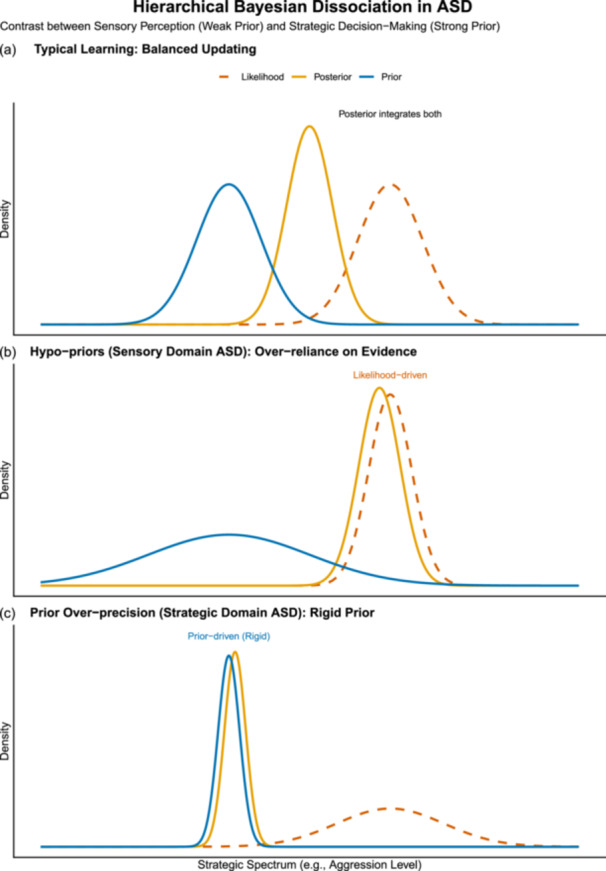
Hierarchical Bayesian dissociation in autism spectrum disorder (ASD): contrast between sensory perception and strategic decision‐making. The conceptual plots illustrate three distinct modes of Bayesian updating, where the prior (blue solid line) and likelihood (red dashed line) are integrated to form the posterior (orange solid line). (a) Typical learning: in neurotypical individuals, the posterior is formed by a balanced integration of internal expectations (prior) and environmental evidence (likelihood), allowing for flexible adaptation. (b) Hypo‐priors (sensory domain ASD): consistent with established models of ASD in sensory perception, a weak prior leads to an over‐reliance on momentary sensory evidence. The posterior is heavily shifted toward the likelihood, potentially explaining sensory hypersensitivity and a lack of contextual modulation. (c) Prior over‐precision (strategic domain ASD): in contrast to the sensory domain, our findings in a complex strategic task (poker) suggest a prior over‐precision model. Here, the internal strategic template (e.g., rigid aggression or avoidance) is represented by an extremely precise (narrow) prior. This results in a posterior that remains anchored to the prior, largely ignoring environmental feedback (likelihood). This computational rigidity explains the observed sex‐specific strategic persistence despite continuous losses against the Game Theory Optimal (GTO) agent.

To bridge the gap between poker behavior and Bayesian inference, we conceptualize the task as a dynamic belief‐updating process.^35^, ^36^ In this framework, prior information represents the participant's internal strategic drive shaped by hand strength,^34^, ^35^, ^50^ while the likelihood consists of environmental evidence revealed during gameplay, such as community cards and opponent actions.^35^, ^50^ Finally, posterior updating is manifested as dynamic strategic shifts, such as capitalizing on hand‐strengthening community cards^50^ or executing strategic resets following a major loss. By analyzing both high‐risk bluffing, which reflects a failure to update during vulnerability, and under‐betting, representing a failure to update during strength, we capture the pervasive nature of prior over‐precision in ASD. Our findings extend existing Bayesian theories of ASD, such as the aberrant precision^30^ model or HIPPEA^29^ model, which is defined as the High, Inflexible Precision of Prediction Errors in Autism. While those models focus on the perceptual domain—positing an over‐reliance on bottom‐up sensory details—our results suggest a distinct profile at the strategic level. In noisy, recursive social environments like poker,^35^ individuals with ASD may default to an inflexible, rule‐based prior, such as system‐driven aggression or safety‐first tendencies, as a compensatory mechanism. This rigid updating points toward a potential hierarchical divergence: while ASD perception may be characterized by weak priors in the sensory domain (Figure 7a,b),^51^, ^52^ ASD strategic decision‐making is defined by over‐precise priors in the cognitive domain (Figure 7c), where negative feedback is treated as noise rather than a signal for belief‐updating.

Amidst these biases, IQ emerged as a critical modulator of likelihood weighting, enabling defensive resets through evidence‐based folding. Higher IQ was associated with a reduction in under‐betting and a more sophisticated style of play. This was marked by fewer passive actions that are easily read by opponents,^53^ alongside a higher frequency of strategic folds. These findings suggest that superior cognitive resources enable players to more accurately weigh environmental evidence. However, a crucial Sex × ADOS‐2 interaction defines the boundary of this compensation. In females, IQ robustly drove strategic resets regardless of trait severity. In contrast, for high‐trait males, the prior over‐precision associated with their aggressive system was so dominant that it effectively neutralized the compensatory effects of IQ, leading to a state of intellectualized rigidity.

The standardized nature of the GTO agent's behavior, combined with the observed stability of strategic patterns over 100 hands, points toward a prior‐driven interpretation. In this view, environmental feedback is filtered to match internal models. This was further evidenced by our sensitivity analyses of the latter half of the session, which demonstrated that the Sex × ADOS‐2 interactions for both high‐risk bluffing and under‐betting remained robust. Intriguingly, advancing age was associated with fewer routine folds and post‐loss resets. This age‐related shift likely reflects a general emotional habituation to financial fluctuations, where older individuals become less sensitive to acute threat or loss under uncertainty. Crucially, because these age effects were statistically controlled as independent covariates, they do not confound our primary findings regarding ASD‐specific rigidity.

It should be noted that in the lower range of ADOS‐2 scores near zero, the predicted curves occasionally show a pattern where females appear more aggressive than males. However, this trend should be interpreted with caution. Since our recruitment primarily focused on individuals with a clinical diagnosis or high autistic traits, the sample size in this near‐typical range was limited, meaning these specific predicted values represent a statistical extrapolation rather than a robust behavioral observation.

This study has several limitations. First, as a pilot investigation, the small sample size may have limited our statistical power to detect associations between certain behaviors and autism indicators, such as the cumulative chip balance. Second, due to the system design, our raw data lacked private hole‐card information for folded hands. Because we could not analyze these folded trials, we were unable to perform a granular strategy analysis using a GTO solver to evaluate the exact distance to mathematically optimal play across all hands, leaving us unable to comprehensively delineate where general affective distress ends and ASD‐specific computational passivity begins. Third, all participants were poker novices; while this ensured a level playing field, their lack of fundamental knowledge, including pre‐flop ranges, positional advantages, or expected value calculations, may have obscured more sophisticated strategic nuances. Fourth, the use of Cepheus, a GTO‐based AI, as the sole opponent may influence the generalizability of our findings to real‐world social interactions. Individuals with ASD often report finding machine‐based interactions more predictable and less anxiety‐inducing than those with humans.^54^, ^55^ Consequently, the strategic rigidity observed here may represent a baseline cognitive phenotype stripped of the social masking or compensatory effort^56^, ^57^, ^58^ typically required in human‐to‐human encounters. While playing against an optimal, non‐reactive AI may reduce emotional engagement^59^ and risk‐taking variance, it concurrently allowed us to observe internal prior precision without the confounding noise of reactive social signals. Fifth, a critical conceptual limitation stems from the absence of a neurotypical control group. Because this study focused exclusively on individuals with elevated autistic traits or clinical diagnoses, it remains unclear whether the observed sexually dimorphic strategic patterns represent ASD‐specific phenotypes or general baseline sex differences in economic decision‐making. Therefore, utmost caution is required before interpreting these results as purely ASD‐driven rigidity. To clarify this specificity, future comparative studies directly contrasting individuals with ASD against neurotypical participants within larger, multi‐opponent cohorts are strictly required.

In conclusion, this pilot study provides the first computational evidence of sexually dimorphic strategic biases in individuals with autistic traits through an incomplete‐information game. Our findings reveal a characteristic divergence in strategic rigidity: while males with higher autistic traits exhibit an aggressive rigidity—manifesting as reckless commitment to weak hands and a failure to adaptively reset after major losses—females demonstrate an avoidant rigidity, characterized by extreme risk‐aversion that persists and even intensifies as the session progresses. We also identified that while IQ functions as a general cognitive resource that facilitates adaptive defensive adjustments, such as strategic folding, it does not mitigate the core strategic biases driven by ASD traits, particularly in high‐risk scenarios. This suggests that the observed rigidities may arise from underlying computational mechanisms, potentially an imbalance in Bayesian belief‐updating, rather than general cognitive deficits. By identifying extreme risk‐aversion as a core computational component of the covert female ASD phenotype, our findings suggest that integrating strategic decision‐making paradigms into clinical frameworks could significantly mitigate ongoing diagnostic delays. Ultimately, these results underscore the necessity of sex‐specific approaches in both the diagnosis and support of individuals on the autism spectrum, paving the way for more nuanced, computationally informed clinical interventions.

## PERMISSION TO REPRODUCE MATERIAL FROM OTHER SOURCES

This article does not make use of any previously published material.

## AUTHOR CONTRIBUTIONS

**Yosuke Eriguchi**: Conceptualization; data curation; formal analysis; funding acquisition; investigation; methodology; project administration; validation; visualization; writing—original draft; writing—review and editing. **Satoshi Eguchi**: Data curation; investigation; resources; writing—review and editing. **Hitoshi Kuwabara:** Project administration; writing—review and editing. **Naoto Aoki**: Writing—review and editing. **Miho Kuroda**: Data curation; investigation; writing—review and editing. **Eisuke Sakakibara**: Writing—review and editing. **Kiyoto Kasai**: Supervision; writing—review and editing. **Yukiko Kano**: Project administration; supervision; writing—review and editing.

## CONFLICT OF INTEREST STATEMENT

The authors declare no conflicts of interest.

## ETHICS APPROVAL STATEMENT

The Ethics Committee of the University of Tokyo Hospital approved this study (Approval number: 12023).

## PATIENT CONSENT STATEMENT

We explained the purpose of this study to all participants, who subsequently provided written informed consent.

## CLINICAL TRIAL REGISTRATION

N/A.

## Supporting information

Supporting File 1.

Supporting File 2.

Supporting File 3.

Supporting File 4.

Supporting File 5.

Supporting File 6.

## Data Availability

The data that support the findings of this study are available from the corresponding author upon reasonable request. All data supporting this study are included within the manuscript.
