# Supplementary figures and images for: Diametrically opposed sex‐specific effects of autistic traits on risk‐taking in poker
Source: PCN Rep. 2026 Jul 7;5(3):e70372. doi: 10.1002/pcn5.70372 (PMC13338710; doi:10.1002/pcn5.70372)

# Individual Cumulative Profit Trajectories

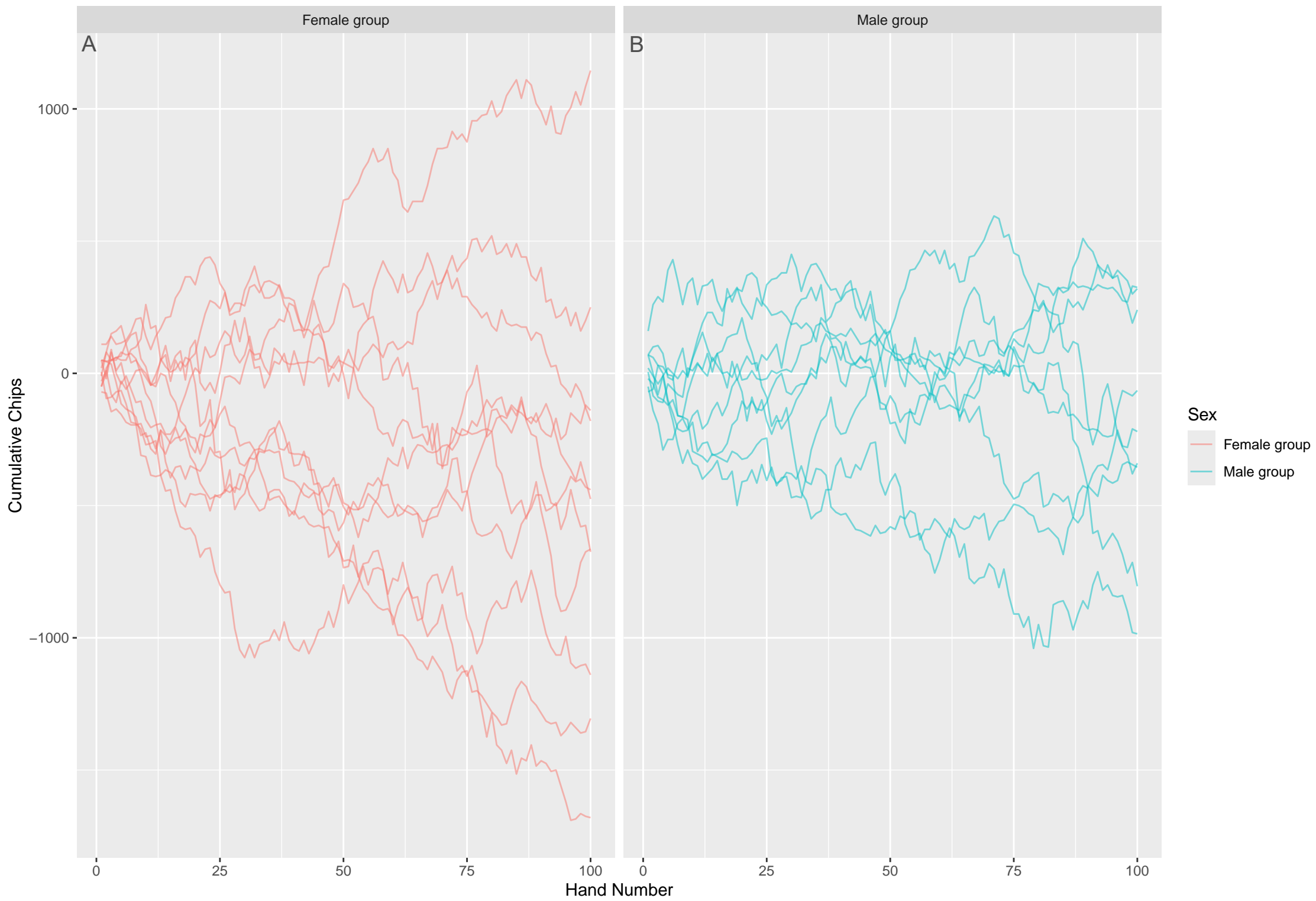

Supplement: Supplementary file 1 — Supporting File 1. [file PCN5-5-e70372-s006.pdf]
