# Supplementary material for: Diametrically opposed sex‐specific effects of autistic traits on risk‐taking in poker
Source: PCN Rep. 2026 Jul 7;5(3):e70372. doi: 10.1002/pcn5.70372 (PMC13338710; doi:10.1002/pcn5.70372)
Supplement: Supplementary file 2 — Supporting File 2. [file PCN5-5-e70372-s002.pdf]

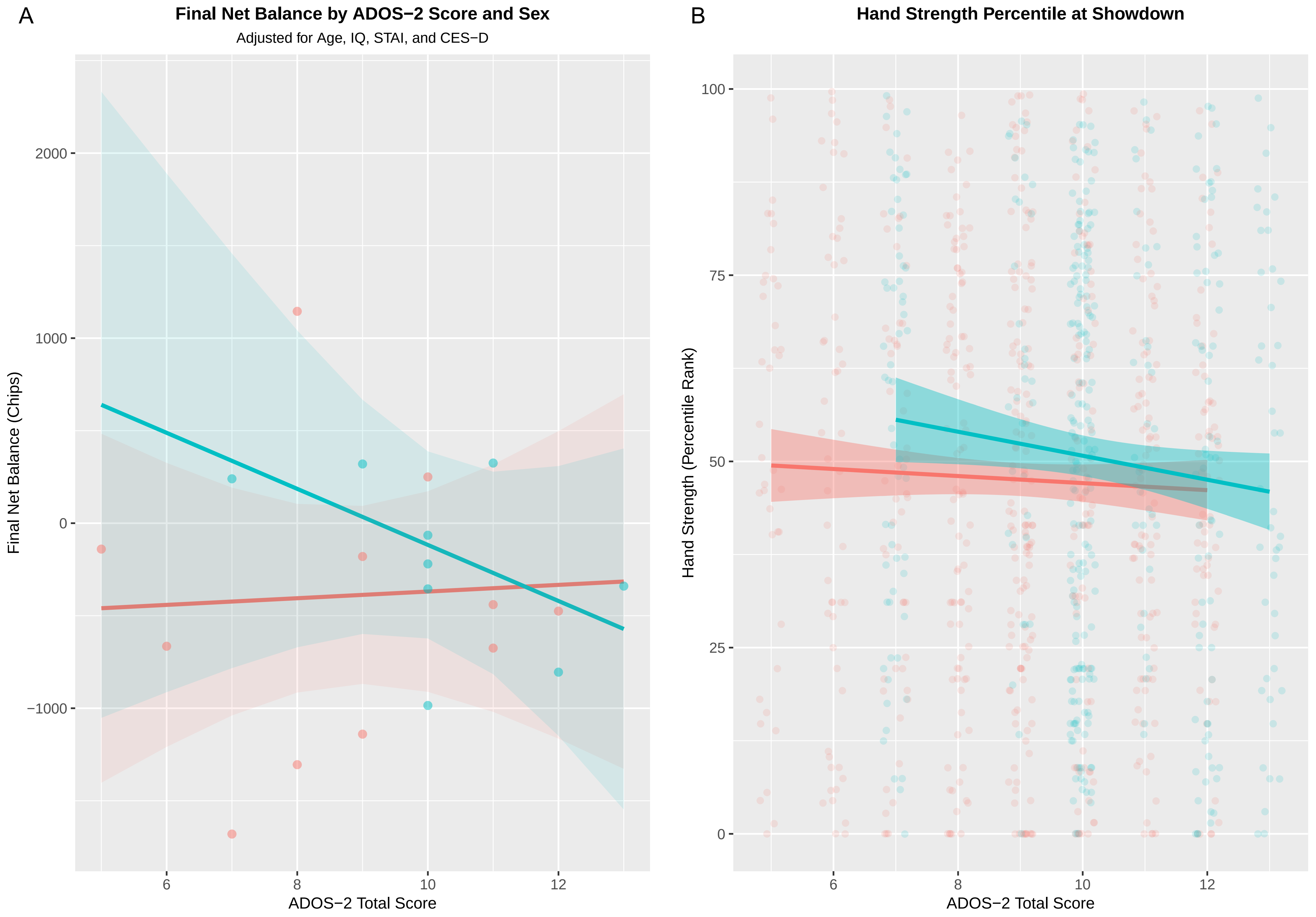
A
Final Net Balance by ADOS−2 Score and Sex
Adjusted for Age, IQ, STAI, and CES−D
Final Net Balance (Chips)
2000
1000
0
−1000
6
8
10
12
ADOS−2 Total Score
B
Hand Strength Percentile at Showdown
Hand Strength (Percentile Rank)
100
75
50
25
0
6
8
10
12
ADOS−2 Total Score
