# Supplementary material for: Diametrically opposed sex‐specific effects of autistic traits on risk‐taking in poker
Source: PCN Rep. 2026 Jul 7;5(3):e70372. doi: 10.1002/pcn5.70372 (PMC13338710; doi:10.1002/pcn5.70372)
Supplement: Supplementary file 6 — Supporting File 6. [file PCN5-5-e70372-s005.docx]

### **Playing rules of heads-up limit Texas Hold’em**

The objective of the heads-up limit Texas Hold’em is to form a better five-card poker hand by combining the player's two hole cards with the five community cards on the board (Figure 1).

- - 1. **The deal**

* Hole cards: Each player is dealt two private cards face down.

* The flop: Three community cards are dealt face up on the board.

* The turn: A fourth community card is dealt face up.

* The river: A fifth and final community card is dealt face up.

- - 1. **The betting rounds**

Following each phase (except the initial deal), players can:

* Fold: Give up their hand and forfeit any bet.

* Call: Match the highest bet made so far.

* Raise: Increase the current bet.

* Check: Not place a bet if no bet has been made.

In the computational framework of poker, every action represents a strategic decision rooted in Bayesian inference. When a player chooses to Bet or Raise, they are attempting to maximize expected value against the opponent’s posterior distribution while emitting a balanced signal to manipulate the opponent's prior beliefs^1,2^. Conversely, a Call implies that the player’s updated posterior probability of winning exceeds the required pot odds^2^, while a Check defers action to gather more evidence^2^. Finally, a Fold occurs when an opponent’s action provides such a strong likelihood of superior strength that the player’s posterior belief of winning drops below profitable margins^2,3^.

- - 1. **Hand rankings**

Standard poker hand rankings apply. A royal flush is the highest hand ranking, and a high card is the lowest (Figure 2).

- - 1. **Showdown**

A showdown occurs if both players remain after the final betting round. Players reveal their hole cards, and the player with the best five-card hand wins the pot.

#### **Key terms**

* Blind: A forced bet that the two players must post to the dealer’s left before the hand begins.

* Pot: The accumulated amount of money bet during a hand and awarded to the winner.

Players have 60 s to choose an action.

**Supplementary Figure legends**

### **Supplementary Figure 1**

**Individual Cumulative Profit Trajectories**

The plots show cumulative net balances over 100 hands for the (A) Female group and (B) Male group against a GTO opponent. While group means were similar, individual trajectories revealed a clear asymmetry. In the male group (B), most participants' balances converged near zero, as their "aggressive rigidity" (e.g., high-risk bluffing) created high variance where gains and losses frequently offset. Conversely, the female group (A) showed a bifurcated pattern: while one individual achieved high gains, a larger subset exhibited a continuous, non-reversing decline.

### **Supplementary Figure 2**

1. **Final Net Balance by ADOS-2 Score and Sex.**

The plot illustrates the association between autistic traits (ADOS-2 total scores) and the final net balance after 100 hands, adjusted for Age, IQ, STAI, and CES-D. No statistically significant association was observed between ADOS-2 scores and the final net balance for either sex (p = 0.218 for the interaction), indicating that overall financial outcomes did not reliably scale with autistic trait severity in this sample.

1. **Hand Strength Percentile at Showdown**: Scatter plots represent individual showdown data, where the x-axis indicates each participant's ADOS-2 total score and the y-axis shows the hand strength percentile (0–100th) at the point of showdown. Regression lines and shaded areas denote the predicted mean hand strength and 95% confidence intervals derived from a generalized linear mixed model (GLMM). A higher percentile indicates stronger hand quality.

### **Reference**

1. Chen, B. & Ankenman, J. *The Mathematics of Poker*. (ConJelCo LLC, 2006).

2. Korb, K. B., Nicholson, A. & Jitnah, N. Bayesian Poker. Preprint at https://doi.org/10.48550/arXiv.1301.6711 (2013).

3. Southey, F. *et al.* Bayes’ Bluff: Opponent Modelling in Poker. Preprint at https://doi.org/10.48550/arXiv.1207.1411 (2012).
